# Urethral stricture: the oldest urologic disease in 2017

**DOI:** 10.1590/S1677-5538.2017.01.01

**Published:** 2017

**Authors:** Luciano A. Favorito

**Affiliations:** MD, PhD. Professor Associado da Unidade de Pesquisa Urogenital da Universidade do Estado de Rio de Janeiro.; Urologista do Hospital da Lagoa Federal, Rio de Janeiro.

The January-February 2017 issue of the International Braz J Urol presents original contributions with a lot of interesting papers in different fields: Female Urinary Incontinence, Male Urinary Incontinence, Bladder Cancer, Pelvic-Ureteric Junction Stenosis, BPH, Prostate Cancer, Bladder Cancer, Renal Cancer, Testicular Cancer, Penile Cancer, Overactive Bladder Syndrome, Ureteral Obstruction, Pediatric Urology, Interstitial Cystitis and Urethral Stricture. The papers come from many different countries such as Brazil, USA, Turkey, Italy, Belgium, India, China, United Kingdon, Portugal, Taiwan and Serbia, and as usual the editor’s comment highlights some papers. We decided to comment 2 papers about a very usual and challenging topic in urologic practice: The Urethral Stricture.

Doctors Pandian and collegues, from India performed on page 125 an interesting study about the Pre-operative evaluation of pelvic fracture urethral distraction defect by MRI. The authors performed a prospective study with 20 patients with pelvic fracture and urethral distraction using IIEF questionnaire to study the erectile funtion, retrograde urethrogram and micturating cystourethrogram (RGU+MCU) and MRI pelvis and concluded that MRI did not offer signicant advantage over MCU in the subgroup of men with normal erections. The cavernosal injury noted on MRI strongly correlated with ED. Role of MRI may be limited to the subgroup with ED or an inconclusive MCU.

Doctor Kanodia and collegues from India too, described on page 161 an interesting challenging clinical case about a Intraoperative breakage of Sachse’s knife blade during a internal urethrotomy and managed with the help of double J stent removing forceps. The authors concluded that this complication should be kept in mind and instruments should be checked properly by the operative surgeon prior to start the procedure. Retained sharp objects like knife blade in urethra as a result of breakage of Sachse’s knife blade can be managed endoscopically.

Urethroplasty is a procedure that has a high success rate but exists a small group of patients with the chance of multiple interventions: Urethal dilatation, Internal urethrotomy and re-do uretrhoplasty (1). The Internal urethrotomy (IU) has the advantages of an easy, simplicity, speedy and shorty convalescence in treatment of urethral stricture (2). Complications of IU are usually minor, including infection and hemorrhage. Previous studies comparing the IU with the urethroplasty shows that the IU requires further surgery or continued self-dilatation compared with urethroplasty (3, 4). In this number of Int Braz J Urol we observed a rare complication of IU: The breakage of the cold knife and this manipulation by endoscopy. This kind of complication needs to be kept in mind during the IU.

Pelvic fracture urethral distraction defect (PFUDD) may be associated with disabling complications, such as recurrent stricture, urinary incontinence, and the most common complication associated with this condition, the erectile dysfunction (5). In this number of Int Braz J Urol we observed a paper that compare the MRI and VCUG, with the prediction of erectile dysfunction in PFUDD. The three-dimensional imaging modalities provide more comprehensive information regarding the anatomy of urethral diseases (6), but in this preliminary report the MRI do not replace the VCUG in the prediction of erectile dysfuntion.

In this new year of 2017 we are still discussing surgical techniques and diagnostic methods of one of the oldest pathologies known to the urologist. The urethral stricture remains a challenging pathology today.

Luciano A. Favorito, MD, PhD. Professor Associado da Unidade de Pesquisa Urogenital da Universidade do Estado de Rio de Janeiro Urologista do Hospital da Lagoa Federal, Rio de Janeiro Editor Associado da International Braz J Urol
